# Supplementary material for: End Sequence Analysis Toolkit (ESAT) expands the extractable information from single-cell RNA-seq data
Source: Genome Res. 2016 Oct;26(10):1397–410. doi: 10.1101/gr.207902.116 (PMC5052061; doi:10.1101/gr.207902.116)
Supplement: Supplemental Material [file supp_gr.207902.116_Supp_Materials.tar.gz › ESAT-esat_v0.1/doc/html/classumms_1_1core_1_1annotation_1_1_abstract_annotation_collection_3_01_t_01extends_01_annotation_01_4.html]

ESAT: umms.core.annotation.AbstractAnnotationCollection< T extends Annotation > Class Reference


ESAT


- Main Page
- Related Pages
- Packages
- Classes
- Files

- Class List
- Class Index
- Class Hierarchy
- Class Members

All Classes Namespaces Files Functions Variables Enumerator Pages

- umms
- core
- annotation
- AbstractAnnotationCollection< T extends Annotation >

Public Member Functions |
Protected Member Functions |
List of all members

umms.core.annotation.AbstractAnnotationCollection< T extends Annotation > Class Referenceabstract

Inheritance diagram for umms.core.annotation.AbstractAnnotationCollection< T extends Annotation >:

[legend]

Collaboration diagram for umms.core.annotation.AbstractAnnotationCollection< T extends Annotation >:

[legend]

|  |  |
| --- | --- |
| Public Member Functions | |
| int | getBasesCovered (Annotation region) |
|  | |
| double | getTotalCoverage (Annotation region) |
|  | |
| double | getAverageCoverage (Annotation region) |
|  | |
| double | getCount (Annotation region) |
|  | |
| double | getCount (AnnotationList<?extends Annotation > set) |
|  | |
| double | getCount (AnnotationList<?extends Annotation > set, boolean fullyContained) |
|  | |
| CloseableIterator< T > | getOverlappingAnnotations (Annotation region) |
|  | |
| Annotation | collapse (Annotation region, boolean fullyContained) |
|  | |
| WindowScoreIterator< CountScore > | scan (int windowSize, int overlap) |
|  | |
| WindowScoreIterator< CountScore > | scan (Annotation region, int windowSize, int overlap) |
|  | |

|  |  |
| --- | --- |
| Protected Member Functions | |
| WindowProcessor< CountScore > | getCountProcessor () |
|  | |

## Detailed Description

Author
:   engreitz Abstract class for functions common to both AlignmentModel and AnnotationSet. Mostly here for consistency in function between the two classes

Parameters
:   |  |  |
    | --- | --- |
    | <T> |  |

## Member Function Documentation

|  |  |  |  |
| --- | --- | --- | --- |
| Annotation umms.core.annotation.AbstractAnnotationCollection< T extends Annotation >.collapse | ( | Annotation | *region*, |
|  |  | boolean | *fullyContained* |
|  | ) |  |  |

|  |  |  |  |  |  |
| --- | --- | --- | --- | --- | --- |
| double umms.core.annotation.AbstractAnnotationCollection< T extends Annotation >.getAverageCoverage | ( | Annotation | *region* | ) |  |

|  |  |  |  |  |  |
| --- | --- | --- | --- | --- | --- |
| int umms.core.annotation.AbstractAnnotationCollection< T extends Annotation >.getBasesCovered | ( | Annotation | *region* | ) |  |

|  |  |  |  |  |  |
| --- | --- | --- | --- | --- | --- |
| double umms.core.annotation.AbstractAnnotationCollection< T extends Annotation >.getCount | ( | Annotation | *region* | ) |  |

|  |  |  |  |  |  |
| --- | --- | --- | --- | --- | --- |
| double umms.core.annotation.AbstractAnnotationCollection< T extends Annotation >.getCount | ( | AnnotationList<?extends Annotation > | *set* | ) |  |

|  |  |  |  |
| --- | --- | --- | --- |
| double umms.core.annotation.AbstractAnnotationCollection< T extends Annotation >.getCount | ( | AnnotationList<?extends Annotation > | *set*, |
|  |  | boolean | *fullyContained* |
|  | ) |  |  |

|  |  |  |  |  |
| --- | --- | --- | --- | --- |
| WindowProcessor<CountScore> umms.core.annotation.AbstractAnnotationCollection< T extends Annotation >.getCountProcessor | ( |  | ) |  |

protected

|  |  |  |  |  |  |
| --- | --- | --- | --- | --- | --- |
| CloseableIterator<T> umms.core.annotation.AbstractAnnotationCollection< T extends Annotation >.getOverlappingAnnotations | ( | Annotation | *region* | ) |  |

|  |  |  |  |  |  |
| --- | --- | --- | --- | --- | --- |
| double umms.core.annotation.AbstractAnnotationCollection< T extends Annotation >.getTotalCoverage | ( | Annotation | *region* | ) |  |

|  |  |  |  |
| --- | --- | --- | --- |
| WindowScoreIterator<CountScore> umms.core.annotation.AbstractAnnotationCollection< T extends Annotation >.scan | ( | int | *windowSize*, |
|  |  | int | *overlap* |
|  | ) |  |  |

|  |  |  |  |
| --- | --- | --- | --- |
| WindowScoreIterator<CountScore> umms.core.annotation.AbstractAnnotationCollection< T extends Annotation >.scan | ( | Annotation | *region*, |
|  |  | int | *windowSize*, |
|  |  | int | *overlap* |
|  | ) |  |  |

---

The documentation for this class was generated from the following file:

- java/umms/core/annotation/AbstractAnnotationCollection.java


---

Generated on Fri Aug 15 2014 15:11:07 for ESAT by  

 1.8.7
